# Cyclohexyl-Substituted Anthracene Derivatives for High Thermal Stability Organic Semiconductors

**DOI:** 10.3389/fchem.2019.00011

**Published:** 2019-01-23

**Authors:** Yicai Dong, Yuan Guo, Hantang Zhang, Yanjun Shi, Jing Zhang, Haiyang Li, Jie Liu, Xiuqiang Lu, Yuanping Yi, Tao Li, Wenping Hu, Lang Jiang

**Affiliations:** ^1^Shanghai Key Laboratory of Electrical Insulation and Thermal Aging, School of Chemistry and Chemical Engineering, Shanghai Jiao Tong University, Shanghai, China; ^2^Beijing National Laboratory for Molecular Sciences, Key Laboratory of Organic Solids, Institute of Chemistry, Chinese Academy of Sciences, Beijing, China; ^3^University of the Chinese Academy of Sciences, Beijing, China; ^4^College of Chemistry and Material Science, Shandong Agricultural University, Taian, China; ^5^School of Ocean Science and Biochemistry Engineering, Fuqing Branch of Fujian Normal University, Fuzhou, China; ^6^Tianjin Key Laboratory of Molecular Optoelectronic Sciences, Department of Chemistry, School of Science, Tianjin, China

**Keywords:** organic semiconductors, anthracene derivatives, thermal stability, organic field-effect transistors, mobility

## Abstract

A novel p-type organic semiconductor with high thermal stability is developed by simply incorporating cyclohexyl substituted aryl groups into the 2,6-position of anthracene, namely 2,6-di(4-cyclohexylphenyl)anthracene (DcHPA), and a similar compound with linear alkyl chain, 2,6-di(4-*n*-hexylphenyl)anthracene (DnHPA), is also studied for comparison. DcHPA shows sublimation temperature around 360°C, and thin film field-effect transistors of DcHPA could maintain half of the original mobility value when heated up to 150°C. Corresponding DnHPA has sublimation temperature of 310°C and the performance of its thin film devices decreases by about 50% when heated to 80°C. The impressing thermal stability of the cyclohexyl substitution compounds might provide guidelines for developing organic electronic materials with high thermal stability.

## Introduction

Organic field-effect transistors (OFETs) with organic semiconductors as key elements have been extensively studied and believed to play a prominent role in future organic electronics, such as flexible displays (Tang et al., 2009), the radio frequency identity tags (RFID) (Subramanian et al., 2005), and various sensors (Huang et al., 2008, 2017; Knopfmacher et al., 2014; Lu et al., 2017b). High mobility and great stability in ambient conditions are two prerequisites of organic semiconductors for high-performance OFETs. In recent years, remarkable progress has been achieved in improving mobility by design and synthesis of new molecules. In this process, it was found that, tailoring molecular structures is quite effective in tuning the molecular packing motifs and energy levels, which meanwhile influences the field-effect performance (Dong et al., 2010; Watanabe et al., 2012; Mori et al., 2013). On the other hand, great efforts have been devoted to improving the stability of OFETs, including environment stability (air and photo stability; Liu et al., 2015a), operating stability, and stability in some extreme conditions like under high temperatures.

Among the diverse organic semiconductors developed so far, anthracene derivatives (Meng et al., 2006; Klauk et al., 2007; Jiang et al., 2008a,b, 2009; Liu et al., 2009, 2016; Li et al., 2015; Chen et al., 2017, 2018) generally exhibit high environment stability and are supposed to have more commercial potential compared with the benchmark material of pentacene, but the thermal stability of anthracene derivatives have rarely been addressed. Usually organic devices are prone to thermal degradation at the elevated operating temperature (Kuribara et al., 2012). Using organic semiconductor with great thermal stability is a straightforward approach for fabricating thermally stable organic transistors. To date, many researches on improving the thermal stability of semiconductors have been reported. For instance, DBTTT, which was derived from DNTT (Park et al., 2015) by replacing the terminal benzene rings of DNTT with two thiophene rings, exhibited good thermal stability with the sublimation temperature above 350°C, and the electrical characteristics of its thin film transistors (TFTs) remained unchanged when elevating the operating temperature from room temperature to 140^o^C. Similarly, another DNTT derivative, namely, DPh-DNTT (Yokota et al., 2013), was designed and corresponding TFTs showed that the mobility of devices decreased by no more than 20% upon annealing to 250^o^C. Despite these advancements, most of them were tested at room temperature after annealing to various temperatures (Sekitani et al., 2004; Yi et al., 2016; Seifrid et al., 2017). There is very few research on the thermal stability of simultaneous testing an OFET during heating (Fan et al., 2018; Gumyusenge et al., 2018), let alone possible strategies for improving the thermal stability of OFETs under elevated testing temperatures.

In this study, we developed a novel p-type organic semiconductor 2,6-di(4-cyclohexylphenyl)anthracene (DcHPA) by simply incorporating cyclohexyl-phenyl groups into the 2,6-position of the molecular skeleton of anthracene, and corresponding *n*-hexyl-phenyl substituted compound 2,6-di(4-*n*-hexylphenyl) anthracene (DnHPA) was also studied for systematical comparison. It was found that, the DcHPA showed sublimation temperature of 360**°**C (5% weight loss) and only 8% performance degradation for DcHPA thin film FETs when heated to 80**°**C, and it still maintained half of its original value up to about 150**°**C. DnHPA showed 50**°**C lower sublimation temperature, and the mobility of DnHPA TFTs decreased by 50% when heated to 80^o^C, then rapidly degraded, and failed to work at 120**°**C. Moreover, single crystal OFETs of DcHPA exhibited an average mobility of 1.98 cm^2^V^–1^s^–1^ and maximum mobility up to 3.16 cm^2^V^–1^s^–1^, which was comparable to that of DnHPA (Xu et al., 2016). All these results indicate that the cyclohexyl-phenyl substitution obviously improves the thermal stability without sacrificing the mobility, and it might provide guidelines for the design and investigation of high-performance semiconductors with high thermal stability in the future.

## Experimental

### Materials and Characterization

All reagents and chemicals were obtained from commercial resources and used without further purification.

**Scheme 1:**
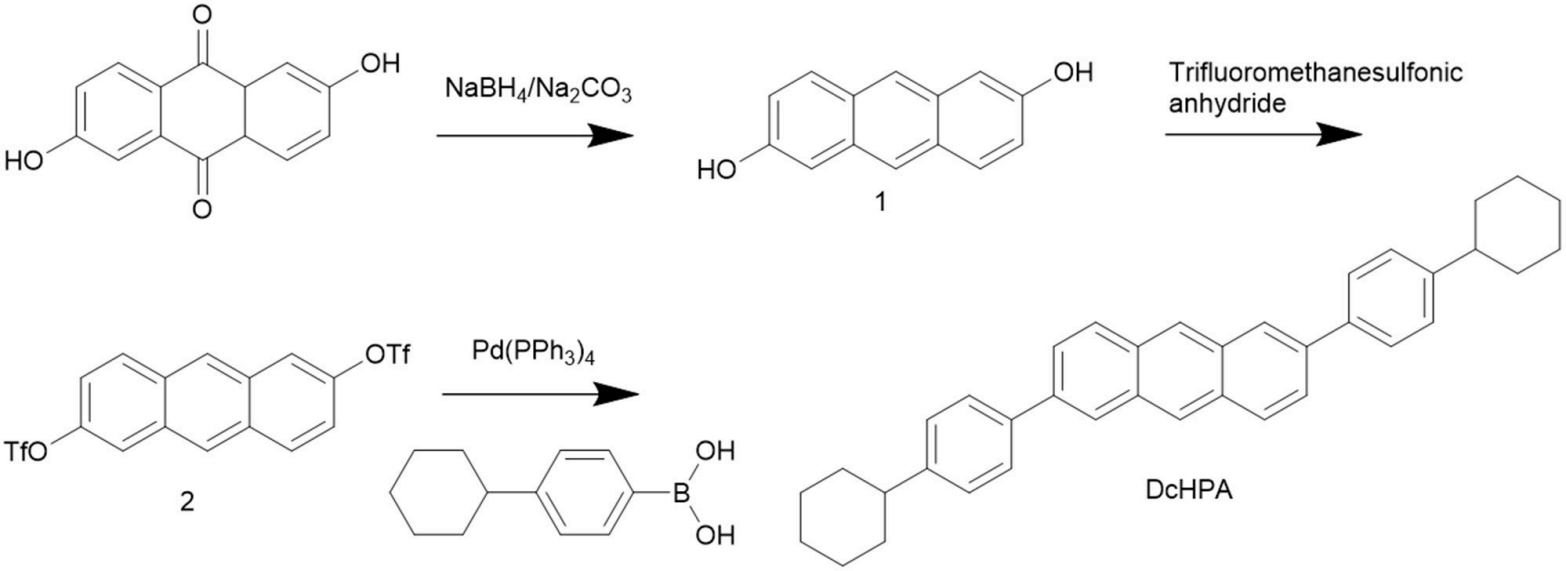
Synthetic route to 2,6-di(4-cyclohexylphenyl)anthracene (DcHPA).

The synthesis of the DcHPA is illustrated in Scheme 1. The final product can be easily synthesized through three simple steps with high yields (Lu et al., 2017a). Firstly a reduction reaction was carried out getting the intermediate of 2,6-dihydroxyl anthracene, and then esterification reaction was done getting 2,6-diyl bis (trifluoromethanesulfonate) anthracene as a yellowish-white crystalline powder, finally by coupling the intermediate 2 with (4-cyclohexylphenyl) boronic acid under the presence of Pd(PPh_3_)_4_ gave the target molecular DcHPA with a yield of 84%. The compound was confirmed by MS (EI): m/z 494 (M^+^), NMR (Figure S1) and elemental analysis calculated for C_38_H_38_ (%): C 92.26, H 7.74. Found: C 91.96, H 7.74.

^1^HNMR spectra were recorded on Advance 400 MHz spectrometer in deuterated chloroform with tetramethylsilane (TMS) as an internal reference. All chemical shifts were reported relative to TMS at 0.0 ppm. Elemental analysis was carried out by Flash EA 1112. The UV-vis spectra were obtained on a Jasco V-570 UV-vis spectrometer with solution concentration of 1 × 10^−5^ mol/L. Cyclic voltammetry (CV) was run on a CHI660C electrochemistry station in THF solution using tetrabutylammonium hexafluorophosphate (Bu_4_NPF_6_) as electrolyte at a scan speed of 100 mVs^−1^, and glassy carbon was used as the working electrode and Pt wire as the counter electrode and ferrocene as inner standard. Thermogravimetric analysis (TGA) was carried out on a Perkin Elmer TGA7 under nitrogen. Differential scanning calorimetry (DSC) was carried out on DSC 250 under nitrogen with the heating rate of 10°C/min. X-ray diffraction (XRD) measurement was performed in reflection mode at 40 kV and 200 mA with Cu Ka radiation using a 2 kW Rigaku D/max-2500 X-ray diffractometer. Atomic force microscopy (AFM) images of single crystal were obtained by using a Digital Instruments Nanoscope III atomic force microscope in air. Transmission electron microscopy (TEM) and selected area electron diffraction (SAED) measurements were carried out on a JEM 1011 (Japan). HR-AFM images were obtained by the instrument of Asylum Research AFM.

### Thin Film Transistors, Fabrication and Characterization

The films were imaged in air using a Digital Instruments Nanoscope III atomic force microscope operated in tapping mode. Thin film devices were fabricated with bottom-gate top-contact configuration. Fifty nanometer thin films of DcHPA and DnHPA were grown on the octadecytrichlorosilane (OTS)-treated Si/SiO_2_ (300 nm) substrate by vacuum deposition with the speed of 0.1–0.4 Å/s at different substrate temperature (T_sub_). Twenty nanometer thick gold source and drain electrodes were deposited successively using the shadow masks with width-to-length ratio (W/L, 240/30 μm) of cal. 8/1. OFET characteristics of DcHPA were obtained in air under different temperatures on a Keysight 1,500 A and Signatone 1,160 probe station, and the heating temperature was varied from 20 to 220^o^C in steps of 20^o^C. Then the mobility was calculated by using the equation: $I_{DS}=\left(W/2L\right)\times C_{i}\times u\times {\left(V_{G}-V_{T}\right)}^{2}$ where W and L are the width and length of channels, respectively, and *Ci* is the capacitance of the gate-dielectric capacitance per unit area.

### Single Crystal Transistors, Fabrication and Characterization

High quality ribbon-like single crystals were grown on OTS-treated Si/SiO_2_ substrate by physical vapor transport (PVT) method in a horizontal tube furnace under argon atmosphere (Wang et al., 2018). In a tube furnace, DcHPA was placed in a quartz boat at the high temperature zone of 180^o^C, and ribbon-like single crystals could be obtained on substrate down the argon stream at the low temperature. The temperature of the tube furnace was gradually increased to 180^o^C, then maintained for 3 h. After cooling down to room temperature, single crystal transistors were fabricated using the “organic ribbon mask” technique (Jiang et al., 2008b).

## Results and Discussions

**Figure 1:**
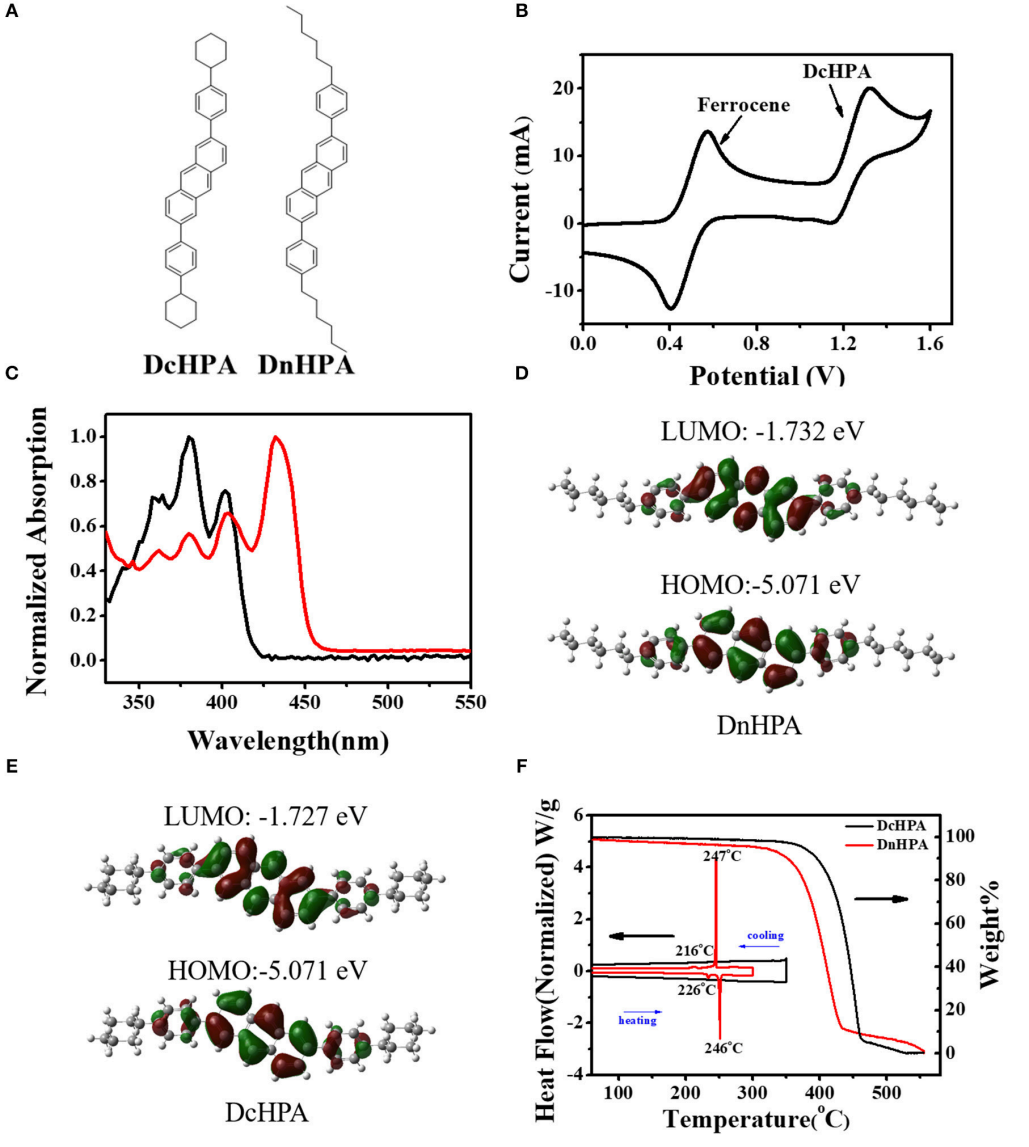
**(A)** Molecular structure of DcHPA and DnHPA. **(B)** Cyclic voltammogram curves of DcHPA in CH_2_Cl_2_ with concentration of 10^−3^ M. **(C)** Uv-vis absorption of DcHPA in dilute CH_2_Cl_2_ solution (10^−5^ M, black) and in thin film state (red). Frontier molecular orbitals for DnHPA **(D)** and DcHPA **(E)**. **(F)** Thermogravimetric analysis (TGA) and differential scanning calorimetry (DSC) of DcHPA and DnHPA.

Figure 1A shows the molecular structure of DcHPA and DnHPA. Cyclic voltammetry measurement of DcHPA was performed in dichloromethane solution (with 10^−3^ M, BuNPF_6_ as electrolyte) to investigate the electrochemical properties. As shown in Figure 1B, it exhibited one quasi-reversible oxidative wave. The highest occupied molecular orbital (HOMO) energy level of DcHPA was calculated from the onset of the oxidation peak with reference to Fc^+^/Fc (4.8 eV) using the equation of E_HOMO_ = [4.8-E_Fc_+E_onsetox_] eV, and HOMO level of 5.6 eV was determined, which was similar to that of 2,6-diphenyl-anthracene (DPA) (Liu et al., 2015a,b). UV-vis measurements of DcHPA in dilute solution (10^−5^ M in CH_2_Cl_2_) and in the form of thin film (50 nm) were performed as shown in Figure 1C. All the spectra of DcHPA exhibited fine vibronic structures of typical anthracene derivatives. The optical bandgap calculated from the onset absorption using the equation of *Eg* = 1, 240/λ, and bandgap of 3 eV was estimated. Besides, frontier orbitals were estimated by theoretical calculation. Firstly, the molecular geometric structures of DnHPA and DcHPA were optimized by density functional theory (DFT) at the B3LYP/6-31G^**^ level. As seen in Figures 1D,E, the electron density distribution of the HOMO and the lowest unoccupied molecular orbital (LUMO) of both DnHPA and DcHPA reside mostly on the central anthracene unit and a bit on the two appended benzene units. The different substituted alkyl groups of n-hexyl and cylco-hexyl hardly affect the HOMO and LUMO energies. Thermal stability of DcHPA and DnHPA was measured by TGA (Figure 1F). The weight of both the materials was almost constant when the temperature was below 300^o^C, and the sublimation temperature (with 5% weight loss) of DcHPA and DnHPA was 360^o^C and 310^o^C under nitrogen atmosphere, respectively. According to results of TGA, DSC measurements were carried out simultaneously to investigate their thermal stability. No endothermic peak and exothermic peak of DcHPA was observed during heating and cooling process before the sublimation temperature, and DnHPA showed endothermic peaks around 226 and 249°C during heating and exothermic peaks around 216 and 245^o^C during cooling in the range of 20 to 300°C. The peaks around 250°C is caused by the melting and crystallization process of DnHPA which was confirmed by the measurement of the melting point, while peaks at around 226 and 216°C might related to a phase transition process.

**Figure 2:**
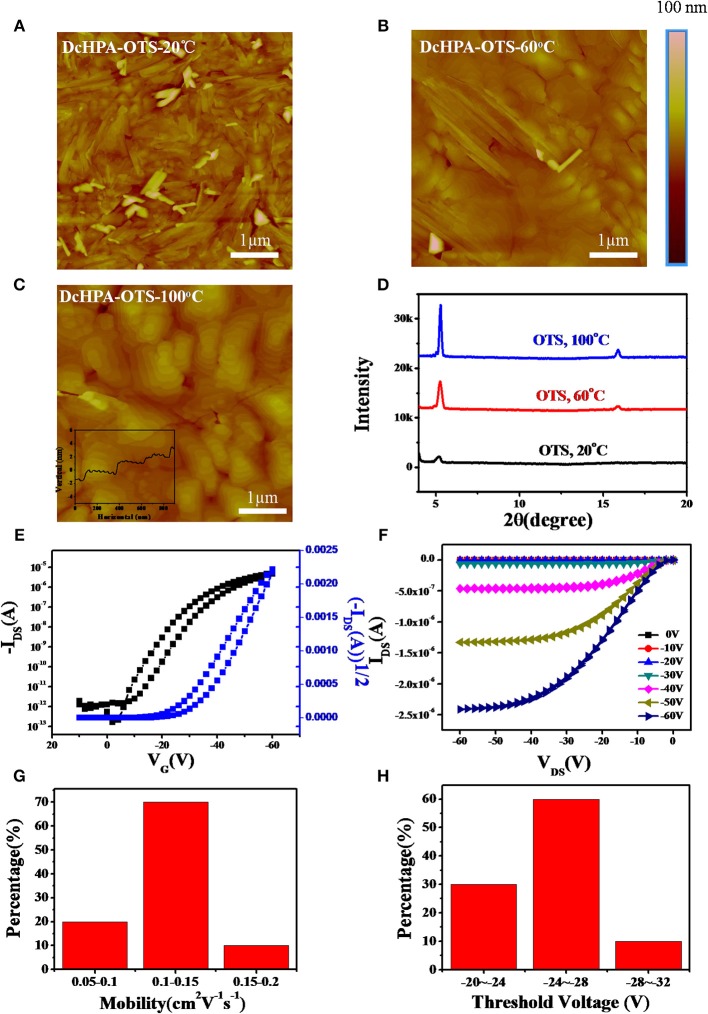
AFM images of 50 nm thin films of DcHPA at **(A)** 20^o^C, **(B)** 60^o^C, and **(C)** 100^o^C, respectively. **(D)** Out of plane XRD results of DcHPA 50 nm films. **(E)** Typical transfer and **(F)** output characteristics of thin film transistors. The distribution of the mobility **(G)** and threshold voltage **(H)** of DcHPA thin film transistors.

DcHPA thin films were deposited on the OTS-treated Si/SiO_2_ substrate with different T_sub_, and the corresponding morphology of thin films were characterized by AFM as illustrated in Figures 2A–C. The films were quite rough and lots of rod-like domains could be observed when deposited on the substrate at room temperature. With the increasing T_sub_, the morphology of DcHPA films gradually became smoother and the rod-like grains gradually evolved into layer-by-layer crystalline domains. Especially in Figure 2C, large and uniform grains with layer-by-layer growth were observed, which proved that increasing the T_sub_ was beneficial to the formation of uniform films. XRD measurements were performed to study the structural order of thin films. Strong and ordered diffraction peaks were obtained (Figure 2D) indicating high crystallinity. Similar diffraction peaks were observed for films deposited at different T_sub_. A primary diffraction peaks appeared at 2θ = 5.30°, with third-order diffraction perks at 2θ = 15.87°. And the same 2θ detected for all these films, indicates the simple morphology change instead of phase transition for thin films deposited at different T_sub_ (He et al., 2015; Zhang et al., 2016). Moreover, the relative high diffraction intensity for films deposited at elevated temperatures indicates higher crystallinity of the films. The d-spacing of DcHPA obtained from the first diffraction peak was 1.67 nm based on the equation of 2*d*×sinθ = *nλ*, which was consistent with the single layer thickness shown in Figure 2C inset. Figures 2E,F showed the typical transfer and output characteristics at room temperature with the T_sub_ of 20^o^C, and slight hysteresis was observed in transfer curves which might be deduced from the trap states in thin films which blocking carrier transport. The distribution of mobility and threshold voltage of 40 devices was shown in the Figures 2G,H, and most devices exhibit mobility in the range of 0.1–0.15 cm^2^V^−1^s^−1^, with the threshold voltage in the range of −24~-28 V. The larger grain size and better crystallinity of films deposited at elevated temperatures also exhibited higher mobility (Table S1), with typical transfer characteristics shown in Figures S2A,B. And the average mobility increased from 0.12 to 0.37 cm^2^V^−1^s^−1^ and 0.52 cm^2^V^−1^s^−1^ for films deposited at T_sub_ from 20 to 60°C and 100°C, respectively. Besides, OFETs studies were also conducted on DnHPA thin films (Figures S3A,B). Typical transfer and output curves were shown in Figures S3C,D, which exhibited mobility of about 0.47 cm^2^V^−1^s^−1^, and thin films deposited at T_sub_ of 60°C showed mobility of 0.54 cm^2^V^−1^s^−1^ (Figure S4A). However, lower mobility of 0.35 cm^2^V^−1^s^−1^ (Figure S4B) was obtained for films deposited at T_sub_ of 100°C. The slightly higher mobility of DnHPA devices than DcHPA devices fabricated at T_sub_ of 20°C could be explained by the larger grain size of DnHPA films (Figures S3A,B) than that of DcHPA thin films (Figure 2A) at the T_sub_ of 20°C, which result in less grain boundaries in the conducting channel. The increased mobility of DcHPA indicated that, the elevated temperature benefited the growth of DcHPA in the whole temperature range. While DnHPA films were found with lots of cracks at T_sub_ of 100°C (Figure S5), which might be caused by the different coefficient of thermal expansion of the semiconductor and the substrates, and these cracks act as traps in the conducting channel and result in the lower mobility.

**Figure 3:**
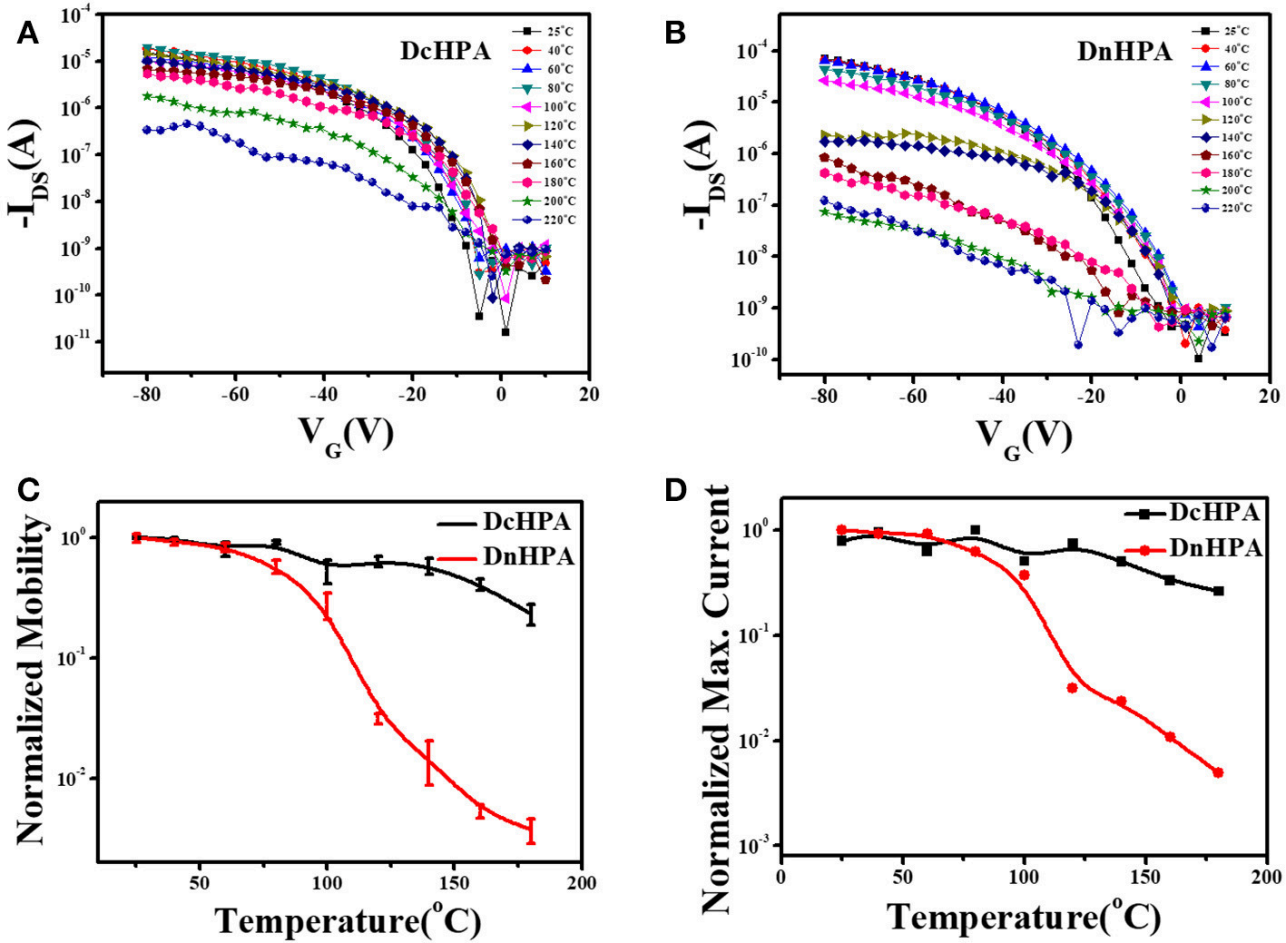
Typical transfer characteristics of DcHPA **(A)** and DnHPA **(B)** at elevated temperature from 20 to 220^o^C in steps of 20^o^C. Field-effect mobility **(C)** and Maximum current **(D)** of DcHPA (black line) and DnHPA (red line) TFTs as a function of heating temperature.

To investigate the thermal stability, OFETs were tested at varied temperatures from 20 to 220^o^C in steps of 20^o^C (Okamoto et al., 2013) for films deposited at T_sub_ of 50^o^C. The AFM images of DcHPA and DnHPA at T_sub_ of 50°C were depicted in Figures S6A,B, respectively. The heating rate was 0.5^o^C/s and the tests were carried out in air during heating. Figures 3A,B showed the transfer characteristics of DcHPA and DnHPA, respectively. Slight performance degradation was observed from room temperature to about 180^o^C for DcHPA devices, and DnHPA devices degraded obviously when heated to 120^o^C. The results were further presented in Figure 3C. The mobility of DcHPA and DnHPA devices as a function of the temperature demonstrated that DcHPA TFTs are significantly more stable than DnHPA TFTs at elevated temperatures. The mobility of DnHPA devices decreased by 50% when heated to 80^o^C, and rapidly degraded thereafter and had almost lost field-effect performance when heated to 120°C. By contrast, the change in mobility of DcHPA devices was as small as 8% when heated to 80^o^C, and the devices still maintained half of its original value up to about 150^o^C. The morphology of thin films at pristine state and after heating were shown in Figures S7A–D, the morphology of DcHPA thin films was almost unchanged, indicating strong thermal stability of them. However, DnHPA thin films became discontinuous after heating and large domains were formed with abundance of cracks, which made poor connectivity of the conducting channel. The change of maximum current in this experiment in Figure 3D was similar to the change of mobility. Obviously, devices of DcHPA have better thermal stability than DnHPA. XRD measurements were also conducted for DcHPA and DnHPA thin films at elevated temperatures from 20 to 200°C in steps of 20°C, and the results were depicted in Figures S8A,B. It can be observed that, there is no significant change of DcHPA thin films in peak position except for slightly changes in intensity. However, the second-order diffraction peak of DnHPA thin films at 2θ = 13.63° obviously disappeared and a shoulder peak emerged at diffraction 2θ = 5.84° at 160°C, and further elevating the temperature resulted in the disappearance of the diffraction at 2θ = 5.46° and intensified diffraction at 2θ = 5.84°, which indicates a phase transition of DnHPA thin films at elevated temperature. And this result correlates well to the DSC result where a phase transition occurs at 226°C. Instead of the phase transition (He et al., 2015; Zhang et al., 2016), quick performance degradation of DnHPA at around 80°C might be caused by the energetic disorder at high temperatures and the deterioration in electrical contacts (Fan et al., 2018).

**Figure 4:**
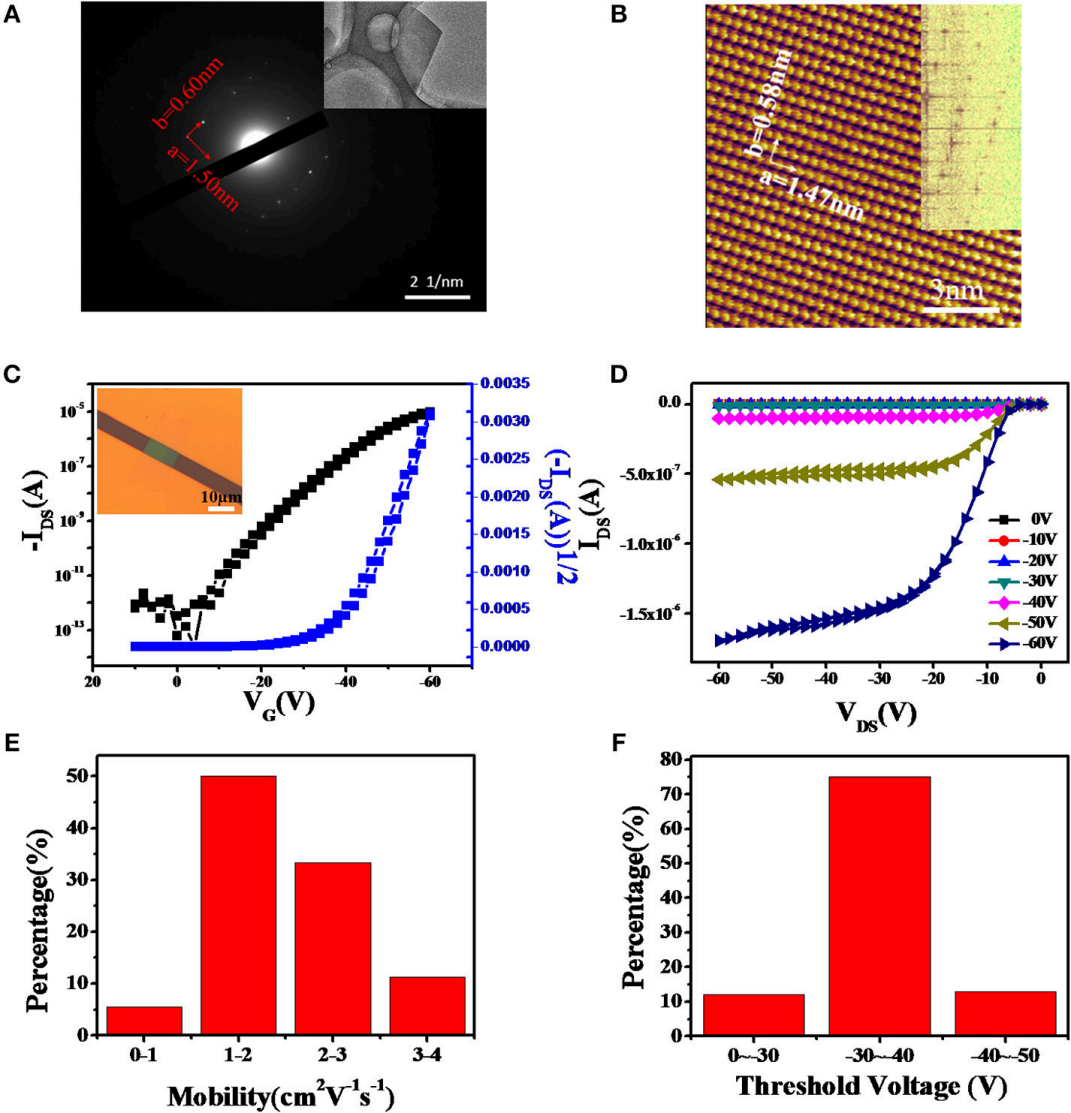
TEM and SAED **(A)** and HR-AFM **(B)** images of DcHPA single crystal obtained by PVT method. **(C)** Typical transfer and **(D)** output characteristics of DcHPA single crystal transistors, and the insert of **(C)** was the optical image of single crystal device based on DcHPA. The distribution of the mobility **(E)** and threshold voltage **(F)** of DcHPA single crystal transistors.

Due to the poor uniformity and small grains of DcHPA thin films, many traps and defects exist in the conducting channel. To address this issue, we next fabricated single crystal transistors to investigate the intrinsic mobility of DcHPA semiconductor. Bottom-gate top-contact single crystal transistors based on DcHPA crystals with thickness of about 20 nm were fabricated using the “organic ribbon mask” technique. Ribbon-like DcHPA microcrystals were obtained by PVT method and AFM image (Figure S9) showed that the DcHPA microcrystals have smooth surfaces and clear edges, which promise HR-AFM characterization (Figure 4B). TEM and SAED were performed for microcrystal grown on copper grid (Figure 4A). And lattice parameters of a-axis and b-axis were about 1.50 nm and 0.60 nm, respectively. HR-AFM image of DcHPA microcrystals was illustrated in Figure 4B. The results showed that the lattice type was rectangular and the lattice parameters of a-axis and b-axis were 1.47 and 0.58 nm, respectively, which were consistent with the SADE results. Combining the results from HR-AFM (Figure 4B and Figure S10D) and XRD (Figure 2D and Figure S3E), the lattice parameters of a = 1.47 nm and b = 0.58 nm with d-spacing of 1.67 nm could be obtained for DcHPA, and the values were 0.44, 0.52, and 1.62 nm for DnHPA, respectively. Since longer molecular length could be expected for the linear DnHPA compared with DcHPA, the comparable d-spacing observed for the two materials, indicating smaller tilt angle for DcHPA in the solid states, which might result in more efficient π-π interactions, and thus enhanced thermal stability and higher mobility. To gain insights into the different thermal stability, a theoretical simulation was carried out regarding 200 molecules in the solid states, however, the intermolecular interaction energy density is almost identical for the two compounds (Supplementary Material theoretical calculation and Figure S11), and further estimation based on the precise packing structure is needed to find the intrinsic origin of the higher thermal stability of DcHPA.

The typical transfer and output characteristics with little hysteresis were illustrated in Figures 4C,D, and the optical image of single crystal transistor structure was shown in the inset in Figure 4C. Mobility of the device was calculated to be 1.83 cm^2^V^−1^s^−1^ by using the equation of $I_{DS}=\left(W/2L\right)\times C_{i}\times u\times {\left(V_{G}-V_{T}\right)}^{2}$ with the channel length of 5.82 μm and channel width of 12.3 μm. A total of 20 devices were measured, and 95% devices exhibited mobility above 1.0 cm^2^V^−1^s^−1^ with the average mobility of 1.98 cm^2^V^−1^s^−1^ as well as the maximum mobility up to 3.16 cm^2^V^−1^s^−1^. The distribution of mobility was shown in Figure 4E. On the other hand, 75% single crystal devices had threshold voltage in the range of −30~-40 V (Figure 4F). And the output in Figure 4D indicated a relatively large contact resistance in the device, which may be highly related to the mismatch between the HOMO energy level of DcHPA (5.6 eV) and the work function of gold (5.1 eV), and the series resistance from the electrode to the conducting channel. Therefore, higher mobility could be expected by reducing the contact resistance (Li et al., 2013). In addition, DnHPA single crystals were obtained and corresponding DnHPA single crystal transistors were fabricated using the same method, which exhibited the average mobility of 1.30 cm^2^V^−1^s^−1^ (Figures S10A–D).

## Conclusions

In summary, we have synthesized a novel semiconductor material DcHPA by simply incorporating cyclohexyl-phenyl groups into the 2, 6-position of the molecular skeleton of anthracene. The effect of deposition temperature on film morphology was explored, showing that the morphology of DcHPA thin films were more uniform, smoother and tended to grow layer-by-layer with higher crystallinity at the elevated substrate temperature. More importantly, thin film devices of DcHPA exhibited apparently higher thermal stability than DnHPA. DcHPA OFETs could maintain half of the original mobility value up to 150^o^C, while the performance of DnHPA thin film devices decreased by 50% when heated to 80^o^C and degraded rapidly thereafter. Although DcHPA and DnHPA have similar molecular weight, DcHPA thin film devices were obviously more stable than DnHPA devices under high temperature. In addition, single crystal transistors were fabricated to investigate intrinsic mobility of DcHPA semiconductor, which exhibits highest mobility of 3.16 cm^2^V^−1^s^−1^. All these results might provide guidelines for the development of materials with high thermal stability.

## Author Contributions

JL, TL, and LJ designed the experiments with help of WH. YD synthesized the compound with help of JL. XL, JL, YD, and HZ designed and performed the thin film and single crystal OFET experiments. JZ and YS helped to obtain TEM, AFM, and HR-AFM characterization. YG and YY helped to obtain theoretical calculation. YD, JL, TL, and LJ wrote the manuscript. All the authors participated in the result discussion.

### Conflict of Interest Statement

The authors declare that the research was conducted in the absence of any commercial or financial relationships that could be construed as a potential conflict of interest.
