# Supplementary material for: Cyclohexyl-Substituted Anthracene Derivatives for High Thermal Stability Organic Semiconductors
Source: Front Chem. 2019 Jan 23;7:11. doi: 10.3389/fchem.2019.00011 (PMC6351495; doi:10.3389/fchem.2019.00011)
Supplement: Supplementary file 1 [file Data_Sheet_1.pdf]

Supplementary Material

Cyclohexyl-substituted anthracene derivatives for high thermal stability organic semiconductors

Yicai Dong[1,2], Yuan Guo[2,3], Hantang Zhang[5,2], Yanjun Shi[2,3], Jing Zhang[2,3], Haiyang Li[2,3], Jie Liu[2*], Xiuqiang Lu[6,2], Yuanping Yi[2], Tao Li[1*], Wenping Hu[4] , Lang Jiang[2*]

[1]Shanghai Key Laboratory of Electrical Insulation and Thermal Aging, School of Chemistry and Chemical Engineering, Shanghai Jiao Tong University, Shanghai 200240, China

[2]Beijing National Laboratory for Molecular Sciences, Key Laboratory of Organic Solids, Institute of Chemistry, Chinese Academy of Sciences, Beijing 100190, China

[3]University of the Chinese Academy of Sciences, Beijing 100039, China

[4]Tianjin Key Laboratory of Molecular Optoelectronic Sciences, Department of Chemistry, School of Science, Tianjin 300072, China

[5]College of Chemistry and Material Science, Shandong Agricultural University, Taian 271018, Shandong, China

[6]Fuqing Branch of Fujian Normal University, Fuzhou, Fujian 350300, China

*** Correspondence:**
Lang Jiang
ljiang@iccas.ac.cn

Tao Li
litao1983@sjtu.edu.cn

Jie Liu
liujie2009@iccas.ac.cn

Synthesis

All reagents and chemicals were obtained from commercial resources and used without further purification.

2,6-Diol-anthracene: To a 100 mL two neck round bottom flask fitted with magnetic bar was added sodium borohydride (2.4 g, 62.5 mmol) and 1M sodium carbonate solution (60 mL). 2,6-dihydroxyanthracene-9,10-dione (1.2 g, 5 mmol) was added in small portions which resulted in colorless gas evolution. After gas evolution stopped, the whole system was heated up to 80 ^{o}C for 30 min. It was then transferred to a 500 mL baker fitted with magnetic bar and slowly acidified with 3M HCl (60 mL) and then filtrated and dried. The filtrate was washed on a filter with acetone and the solution was evaporated under reduced pressure to afford brown solid 0.72 g (68% yield). ^{1}H-NMR (400 MHz, $CDCl_3$): δ [ppm] 9.69 (s, 2H), 8.19 (s, 2H), 7.87 (d, 2H), 7.18 (d, 2H), 7.12 (dd, 2H). MS (EI): m/z 210 (M+).

2,6-Diyl bis(trifluoromethanesulfonate)-anthracene: To a 50 mL two neck round bottom flask charged with magnetic bar was added crude product 1 (420 mg, 2 mmol), then protected under argon, dry dichloromethane (8 mL) and triethylamine (1.4 mL). After cooling to -20 ^{o}C, trifluoromethanesulfonic anhydride (0.82 mL, 5 mmol) was slowly added dropwise via syringe. The mixture was stirred for 2 h and then warmed up to room temperature. Dichloromethane (10 mL) was added and the mixture was washed with water (10 mL ×3) and brine (10 mL ×3). The organic layer was separated, dried over Na_2SO_4 and evaporated under reduced pressure, the crude product was purified by column chromatography (silica, petroleum / dichloromethane) to afford white yellowish solid (550 mg, 58% yield). ^{1}H-NMR (400 MHz, $CDCl_3$): δ [ppm] 8.41 (s, 2H), 8.03 (d, 2H), 7.89 (d, 2H), 7.40 (dd, 2H). MS (EI): m/z 474 (M+).

2,6-di (4-cyclohexylphenyl) anthracene (DcHPA): To a 100 mL flask, 474 mg (1 mmol) 2,6-Diyl bis (trifluoromethanesulfonate)-anthracene, 450 mg (2.2 mmol) (4-cyclohexylphenyl) boronic acid and $Pd(PPh_3)_4$ 63 mg (0.05 mmol) was added under argon. Then 2 mL ethanol, 8 mL toluene and 2 mL 2 M K_2CO_3 aqueous solution was added. The whole system was heated to 90 ^{o}C and kept overnight. Then the whole system was filtered, the filtrate was washed with triethylamine, dichloromethane, water and ethanol successively. DcHPA was obtained as a greenish yellow solid in a yield of 83% (410 mg). And the product was further purified by train-sublimation. ^{1}H NMR (400 MHz, $CDCl_3$): δ[ppm] 8.46, 8.43, 8.18, 8.06, 7.77, 7.75, 7.73, 7.71, 7.52, 7.49, 7.37, 7.35, 2.73, 2.68, 2.60, 2.49, 1.97, 1.94, 1.90, 1.77.MS (EI): m/z 494 (M+). Elemental analysis calculated for $C_{38}H_{38}$ (%): C 92.26, H 7.74. Found: C 91.96, H 7.74

Theoretical calculation

To gain an insight into the intermolecular packing of DnHPA and DcHPA, the thin films were built and imitated by atomistic molecular dynamic simulations with the Gromacs-4.6.7 software package. The simulation procedure is described as follows: (i) randomly placing 200 molecules in a large box (15×15×15 nm^3) to generate an initial structure; (ii) 5 ns NPT equilibration at 600 K and 100 bar to make the molecules close together quickly; (iii) 1.5 ns simulation at 600 K and 1 bar then following by1 ns simulation at 300 K and 1 bar; (iv) 5 ns equilibration at 300K and 1bar. The velocity rescaling thermostat and the Berendsenbarostat under the NPT ensemble were applied to control the temperature and pressure, respectively. Figure S11 shows the molecular packing morphologies. And the intermolecular interaction energy density for DcHPA (394.7 $Jmol^{-1}nm^{-3}$) is almost the same to that of DnHPA (395.2 $Jmol^{-1}nm^{-3}$). As evidenced by the HR-AFM and XRD results, DnHPA is more tilted in the solid states, which will heavily weaken the intermolecular π-π interaction energy, while in the simulation results, the DnHPA molecules are more stretched with strong π-π interaction, these phenomenons are conflict to each other, which calls for more specific estimation based on the precise packing structure.

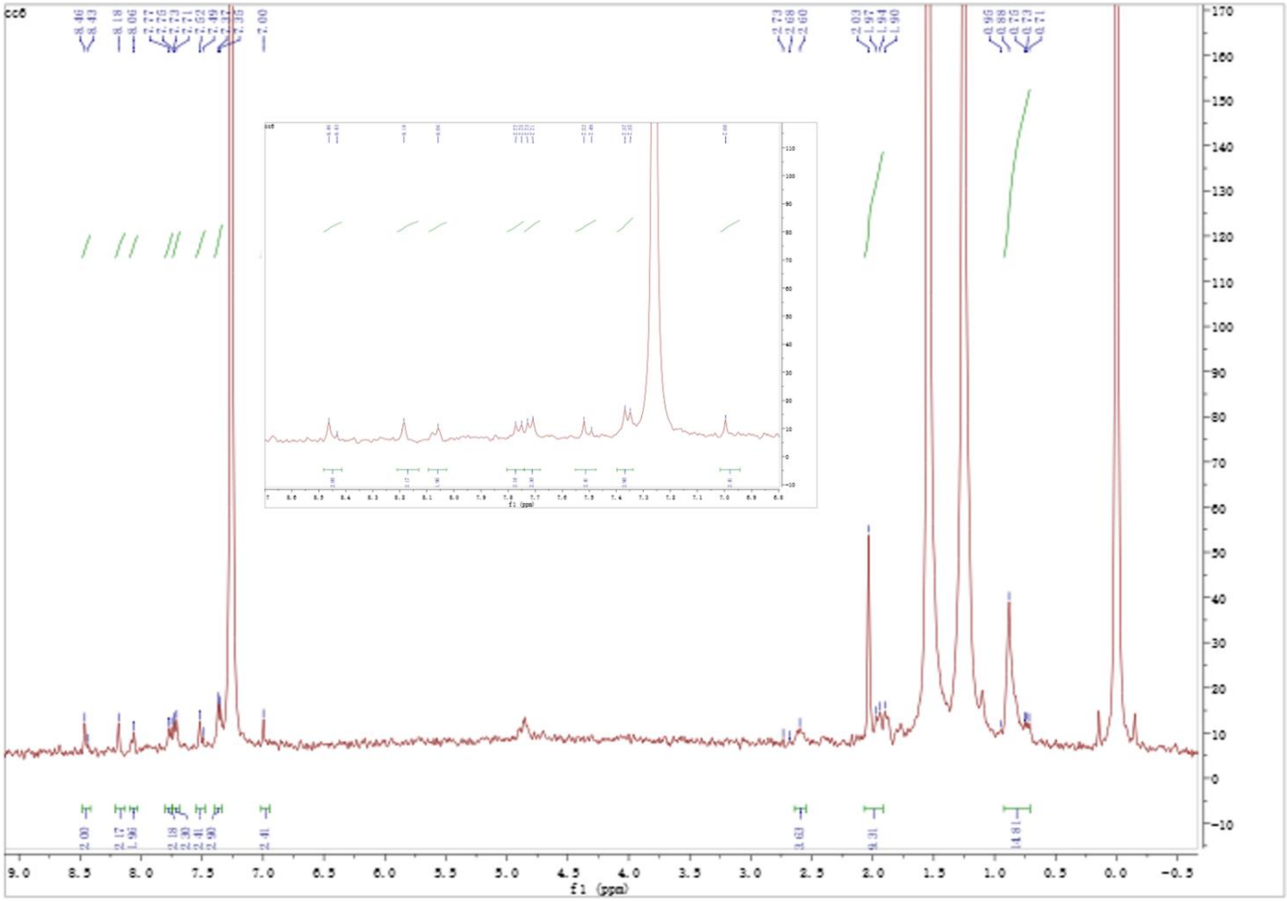

Fig.S1 ^{1}HNMR results of DcHPA in deuterated chloroform.

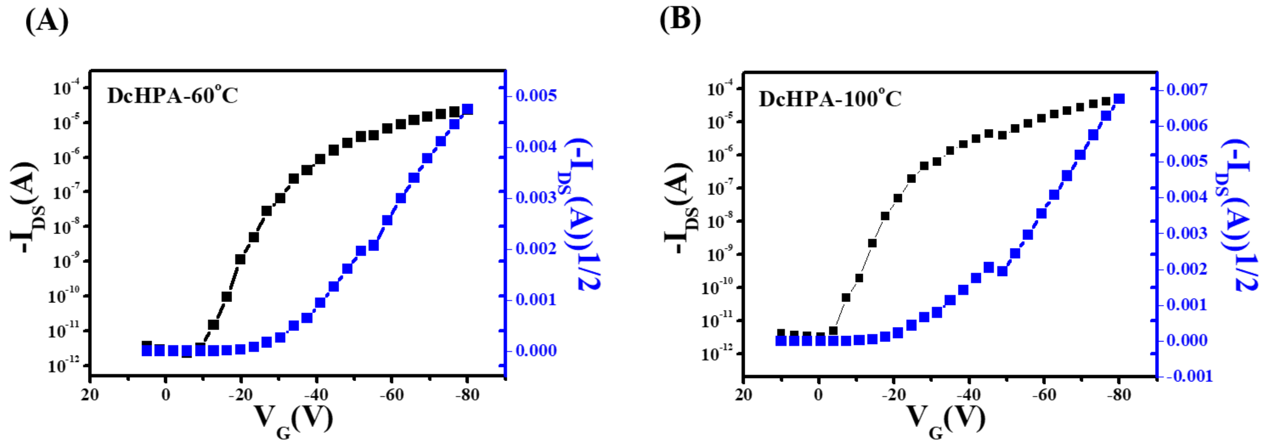

Fig.S2 Typical transfer characteristics of DcHPA thin film transistors with substrate temperature of 60 ^{o}C, 100 ^{o}C, respectively.

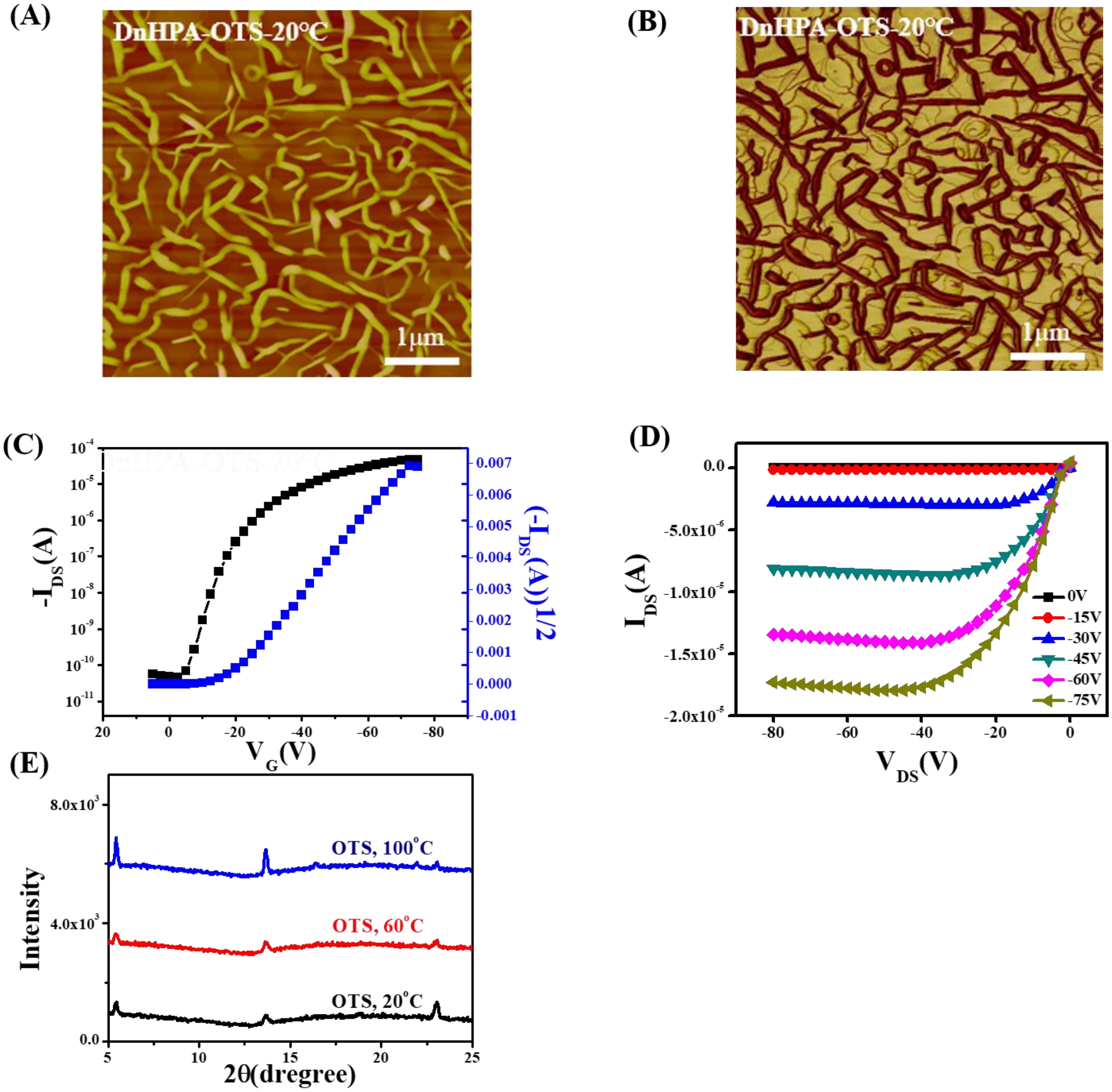

Fig.S3 AFM image (A) and corresponding phase diagram (B) of 50 nm thin films of DnHPA with the substrate temperature of 20 ^{o}C. Typical transfer (C) and output (D) characteristics of DnHPA thin film transistors. (E) Out of plane XRD results of DnHPA 50 nm films.

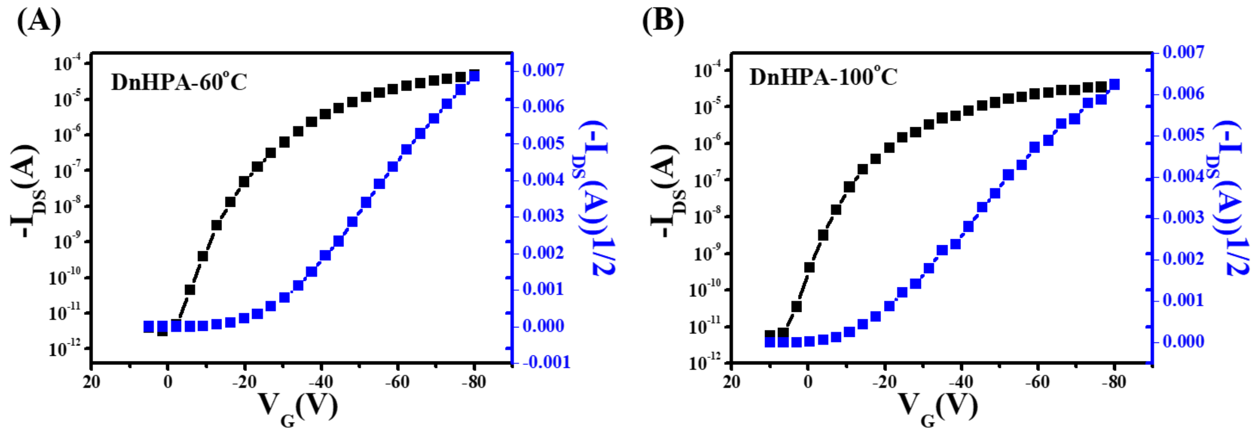

Fig.S4 Typical transfer characteristics of DnHPA thin film transistors with substrate temperature of 60 ^{o}C, 100 ^{o}C, respectively

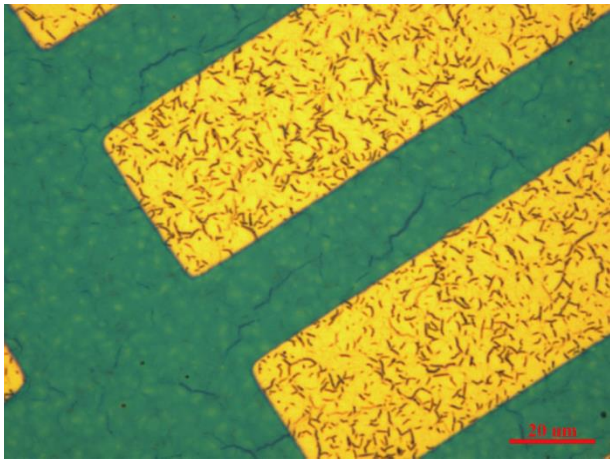

Fig.S5 Optical image of DnHPA thin film devices with the substrate temperature of 100 ^{o}C.

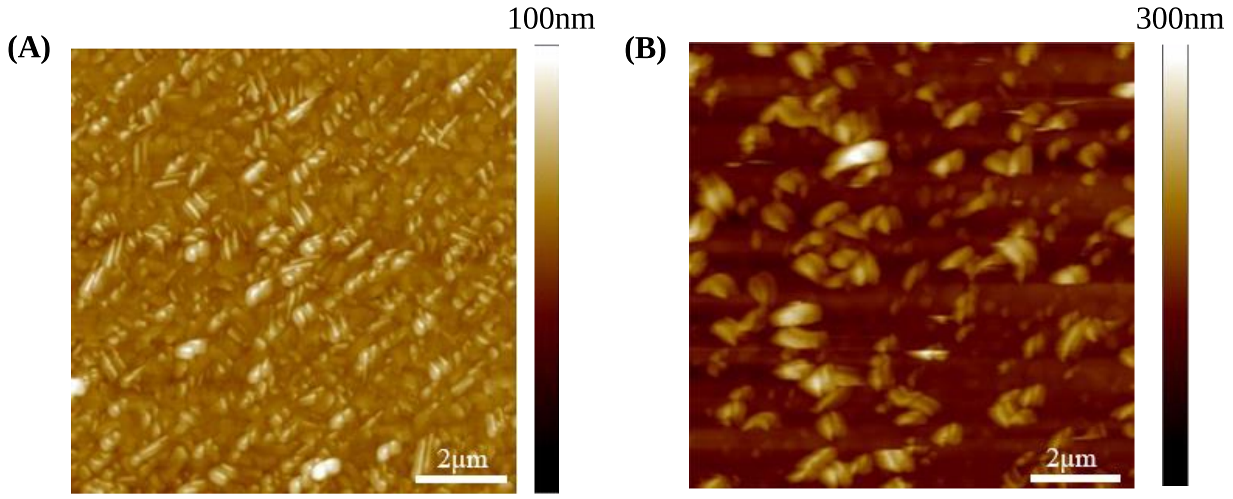

Fig.S6 AFM images of 50 nm thin films of DcHPA (A) and DnHPA (B) with the substrate temperature of 50 ^{o}C.

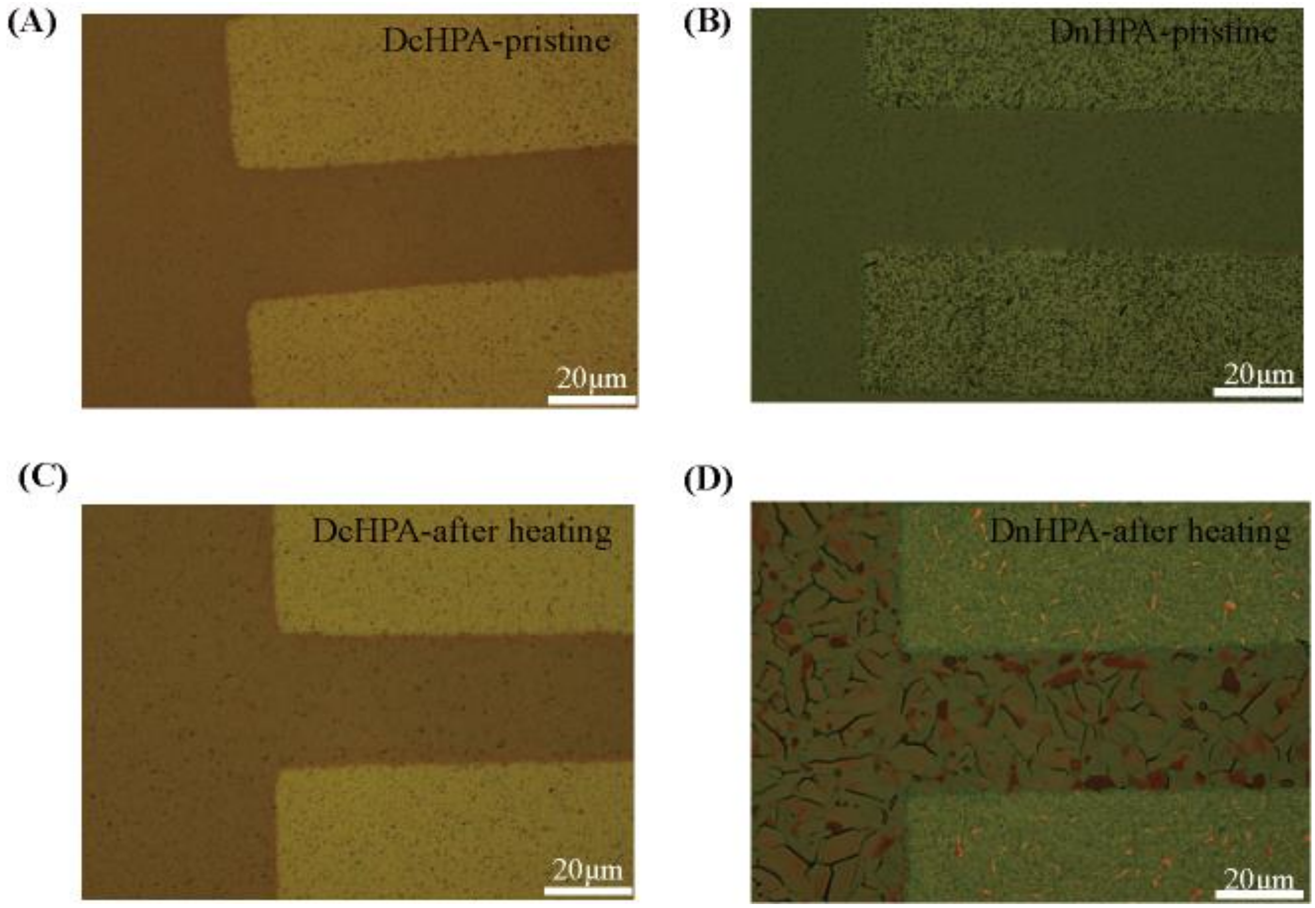

Fig.S7 Optical images of DcHPA and DnHPA thin film devices at pristine state and after heating and testing at 220 ^{o}C.

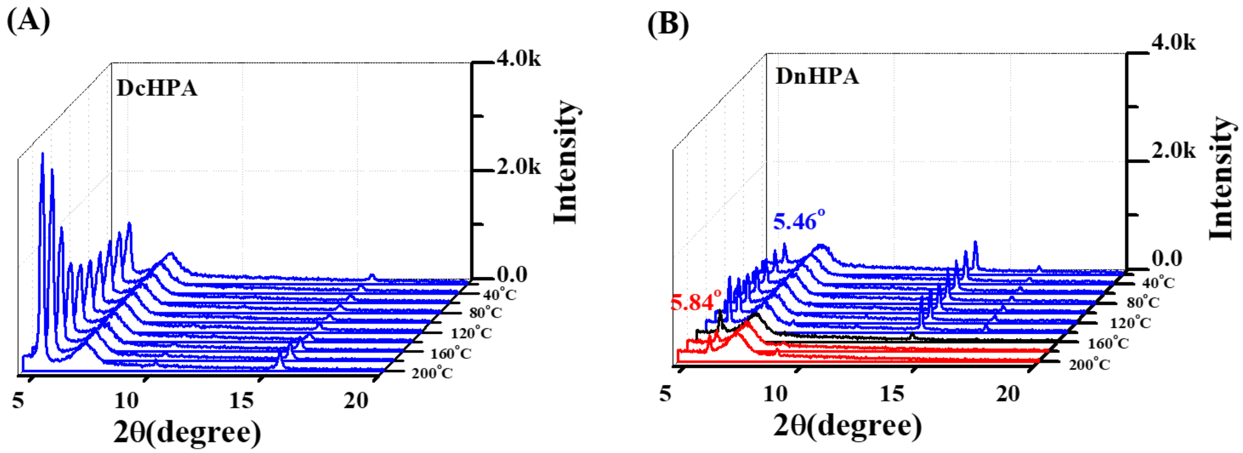

Fig.S8 XRD results of DcHPA (A) and DnHPA (B) thin films at elevated temperature from 20 °C to 220 °C in steps of 20 °C.

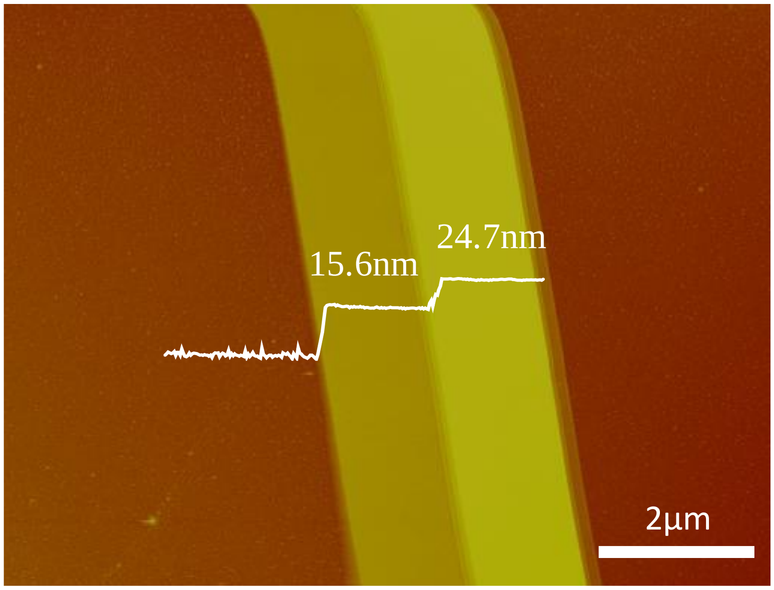

Fig. S9 AFM image of DcHPA single crystal grown on OTS-treated Si/SiO_2 substrate.

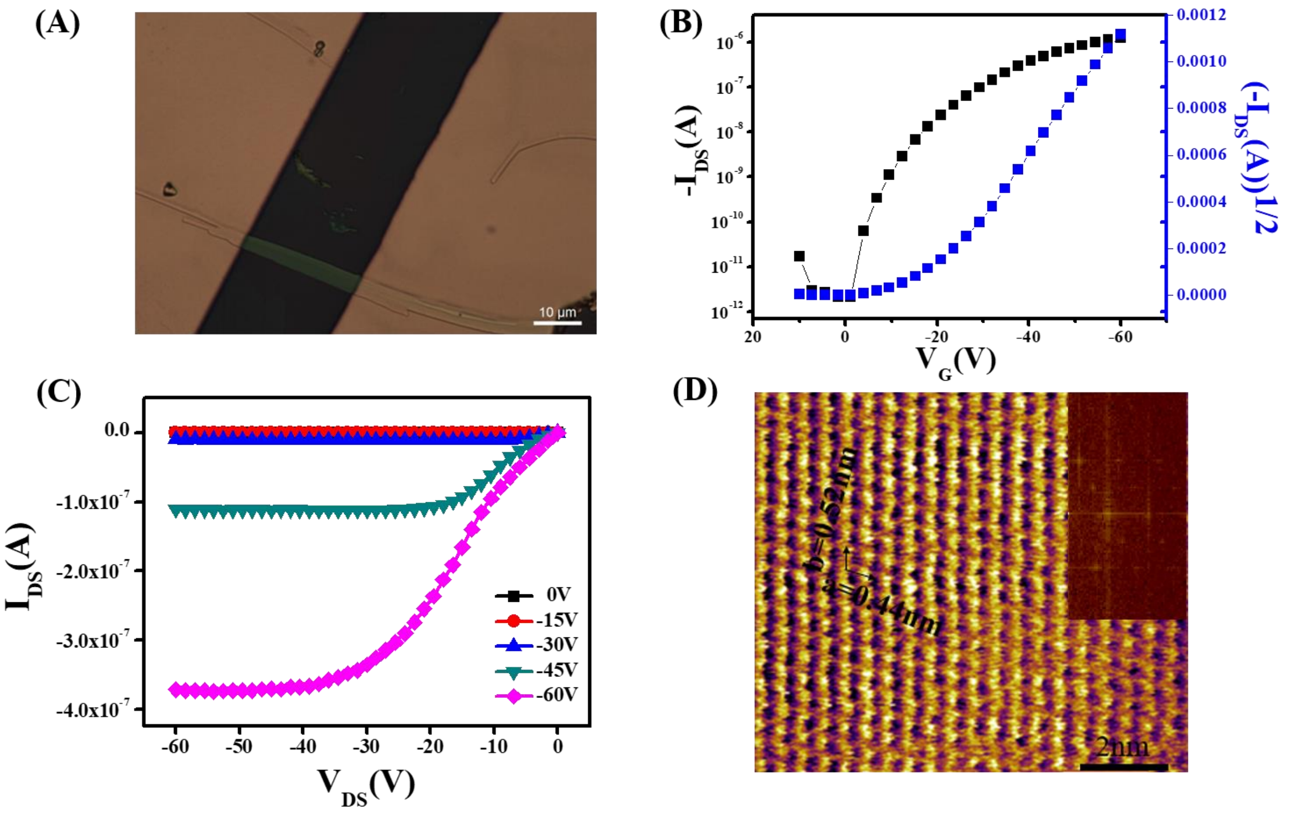

Fig.S10 (A) Optical image of DnHPA single crystal devices. (B) Typical transfer and (C) output characteristics of DnHPA single crystal transistors. (D) HR-AFM image of DnHPA single crystals.

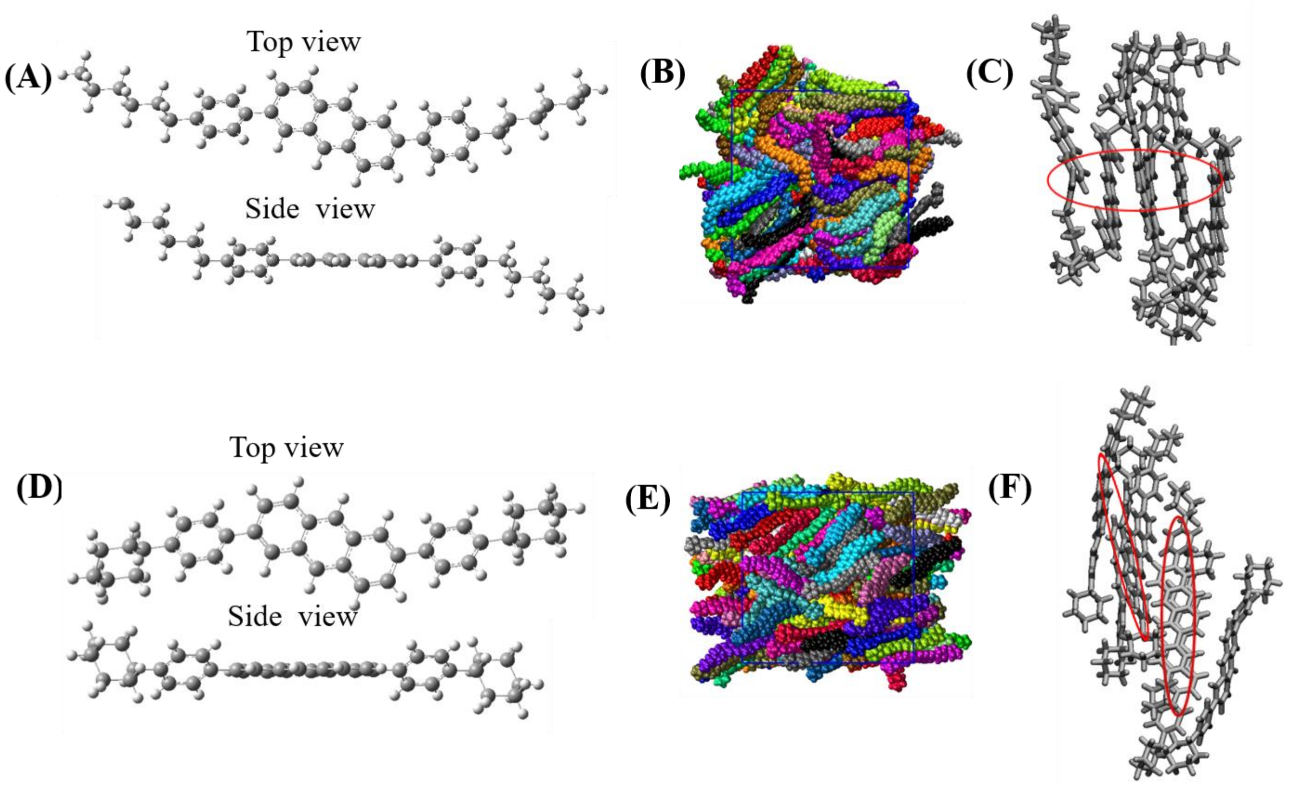

Fig. S11 The optimized molecular geometries of DnHPA and DcHPA (A and D), and molecular packing morphologies based on 200 molecules (B and E). The main intermolecular interactions among the molecules are depicted in (C and F).

Table S1. Performance of vacuum deposited DcHPA thin film devices with substrate temperature of 20 ^{o}C, 60 ^{o}C, 100 ^{o}C, respectively.

Substrate temperature (^{o}C)	Average mobility ($cm^2V^{-1}s^{-1}$)	Threshold voltage (V)
20	0.12	-25
60	0.37	-45
100	0.52	-35
